# High-fat diet-mediated dysbiosis exacerbates NSAID-induced small intestinal damage through the induction of interleukin-17A

**DOI:** 10.1038/s41598-019-52980-2

**Published:** 2019-11-14

**Authors:** Naoki Sugimura, Koji Otani, Toshio Watanabe, Geicho Nakatsu, Sunao Shimada, Kosuke Fujimoto, Yuji Nadatani, Shuhei Hosomi, Fumio Tanaka, Noriko Kamata, Koichi Taira, Yasuaki Nagami, Tetsuya Tanigawa, Satoshi Uematsu, Yasuhiro Fujiwara

**Affiliations:** 1 Department of Gastroenterology, Osaka City University Graduate School of Medicine, 1-4-3 Asahimachi, Abeno-ku, Osaka, 545-8585 Japan; 2 Department of Immunology and Genomics, Osaka City University Graduate School of Medicine, 1-4-3 Asahimachi, Abeno-ku, Osaka, 545-8585 Japan; 3 Division of Innate Immune Regulation, International Research and Development Center for Mucosal Vaccines, The Institute of Medical Science, The University of Tokyo, 4-6-1 Shirokanedai, Minato-ku, Tokyo, 108-8639 Japan; 4 Department of Immunology and Infectious Diseases/Genetics and Complex Diseases, Harvard T. H. Chan School of Public Health, Room 904, Building 1, 665 Huntington Avenue, Boston, Massachusetts, 02115 United States

**Keywords:** Gastroenteritis, Experimental models of disease

## Abstract

Non-steroidal anti-inflammatory drugs (NSAIDs) cause damage in the small intestine in a bacteria-dependent manner. As high-fat diet (HFD) is a potent inducer of gut dysbiosis, we investigated the effects of HFD on bacterial flora in the small intestine and NSAID-induced enteropathy. 16S rRNA gene analysis revealed that the population of *Bifidobacterium* spp. significantly decreased by fold change of individual operational taxonomic units in the small intestine of mice fed HFD for 8 weeks. HFD increased intestinal permeability, as indicated by fluorescein isothiocyanate-dextran absorption and serum lipopolysaccharide levels, accompanied by a decrease in the protein expressions of ZO-1 and occludin and elevated mRNA expression of interleukin (IL)-17A in the small intestine. HFD-fed mice exhibited increased susceptibility to indomethacin-induced damage in the small intestine; this phenotype was observed in normal diet-fed mice that received small intestinal microbiota from HFD-fed mice. Administration of neutralizing antibodies against IL-17A to HFD-fed mice reduced intestinal permeability and prevented exacerbation of indomethacin-induced damage. Thus, HFD-induced microbial dysbiosis in small intestine caused microinflammation through the induction of IL-17A and increase in intestinal permeability, resulting in the aggravation of NSAID-induced small intestinal damage.

## Introduction

Non-steroidal anti-inflammatory drugs (NSAIDs) are the most widely used class of drugs for the treatment of inflammatory conditions and pain management. It is well known that the use of NSAIDs is associated with gastrointestinal damage in aging populations. In addition, several studies using video capsule endoscopy (CE) have demonstrated that NSAIDs can damage the small intestine as well as the upper gastrointestinal tract^1–3^. Although the adverse effects of NSAIDs on the small intestine have been recognized as a healthcare crisis, the understanding of the factors that potentiate NSAID-induced small intestinal damage is limited. Gastric acid is not involved in NSAID-induced small intestinal damage; hence, acid secretion inhibitors, such as proton pump inhibitors (PPIs), cannot provide therapeutic effects against the damage. On the contrary, Wallace *et al*. reported that PPIs alter the composition of rat intestinal bacteria, including the reduction of jejunal Actinobacteria and *Bifidobacteria* spp., which results in the exacerbation of NSAID-induced small intestinal ulcers^4^. Together with the facts that intestinal bacteria play an important role in the pathophysiology of small intestinal damage by their capacity to activate the innate immune system, this microbiome study also offers the possibility that factors affecting the small intestinal flora could aggravate NSAID-induced small intestinal damage.

Abnormality in the composition of the microbial community is known as dysbiosis, and this condition is associated with various diseases, e.g., inflammatory bowel disease^5^, non-alcoholic fatty liver disease^6^, and colorectal cancer^7^. In addition, dysbiosis is closely related to dietary factors, and high-fat diet (HFD) is a potent inducer of dysbiosis which can result in the promotion of obesity^8^. HFD-induced dysbiosis attenuates intestinal barrier function^9^, resulting in leaky gut that causes metabolic endotoxemia, inflammation, and associated disorders due to increased intestinal permeability^10^. Therefore, it is important to accurately evaluate the dietary factors to prevent the NSAID-induced small intestinal damage.

In this study, we investigated changes in bacterial flora and permeability within the small intestine after HFD intake and their influence on NSAID-induced small intestinal damage in mice.

## Results

### Exacerbation of indomethacin-induced small intestinal damage in mice fed HFD

**Figure 1:**
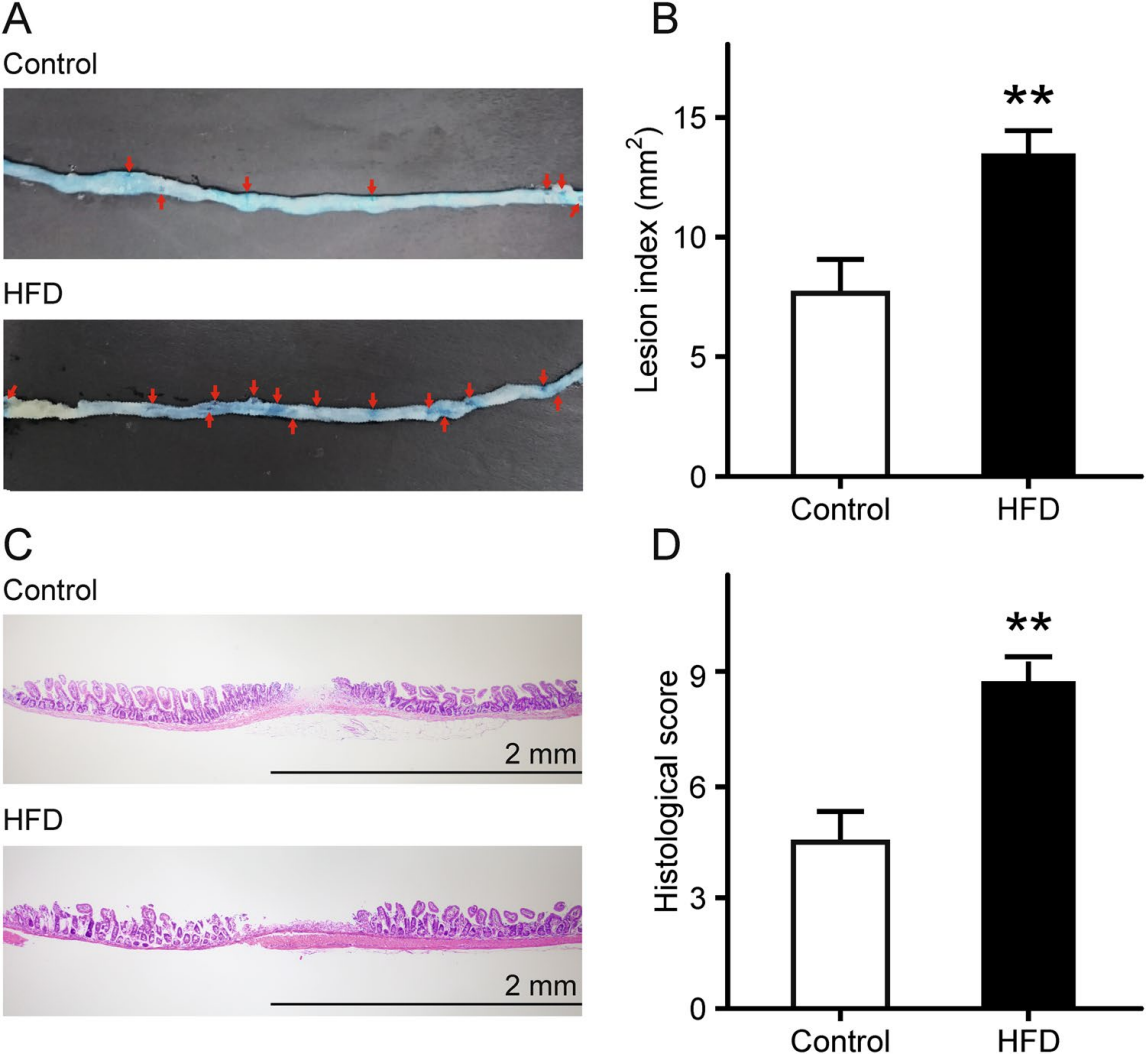
Small intestinal damage after indomethacin administration in HFD-fed mice. (**A**) Damaged mucosa was stained dark blue with 1% Evans blue (arrows). (**B**) Lesion index after administration of indomethacin. The area (mm^2^) of macroscopically visible lesion was measured, summed per small intestine, and designated as the lesion index. n = 7–9. (**C**) Representative histological images of indomethacin-induced small intestinal damage (hematoxylin and eosin stain). (**D**) Histological evaluation of indomethacin-induced small intestinal damage. n = 4–5. Each column represents mean ± standard error of the mean. **p < 0.01 vs. control mice. Control, control group fed AIN-93M; HFD, HFD group fed HFD-60.

Mice were randomly separated into a control group fed AIN-93M (normal diet) or an HFD group fed HFD-60 for 8 weeks. Body weight (BW) was recorded weekly, and BW in the HFD group significantly increased compared with that of the control group (Supplementary Fig. S1). Mice were subsequently administered indomethacin (12 mg/kg) by gavage and sacrificed 24 h later. Lesion index, the summed area (mm^2^) of macroscopically visible lesion as observed with 1% Evans blue staining, increased significantly by 2.0-fold in the HFD group, compared with that in the control group (*p* = 6.4 × 10^−3^; Fig. 1A, B). In addition, histological examination of samples from the HFD group showed intestinal sloughing and destruction of the upper epithelium with infiltration of inflammatory cells as well as mucosal ulceration, which extended into the submucosal layer; these observations suggest relatively severe indomethacin-induced mucosal damage (*p* = 2.5 × 10^−3^; Fig. 1C, D).

### HFD-induced alterations in the small intestinal microbiota

**Figure 2:**
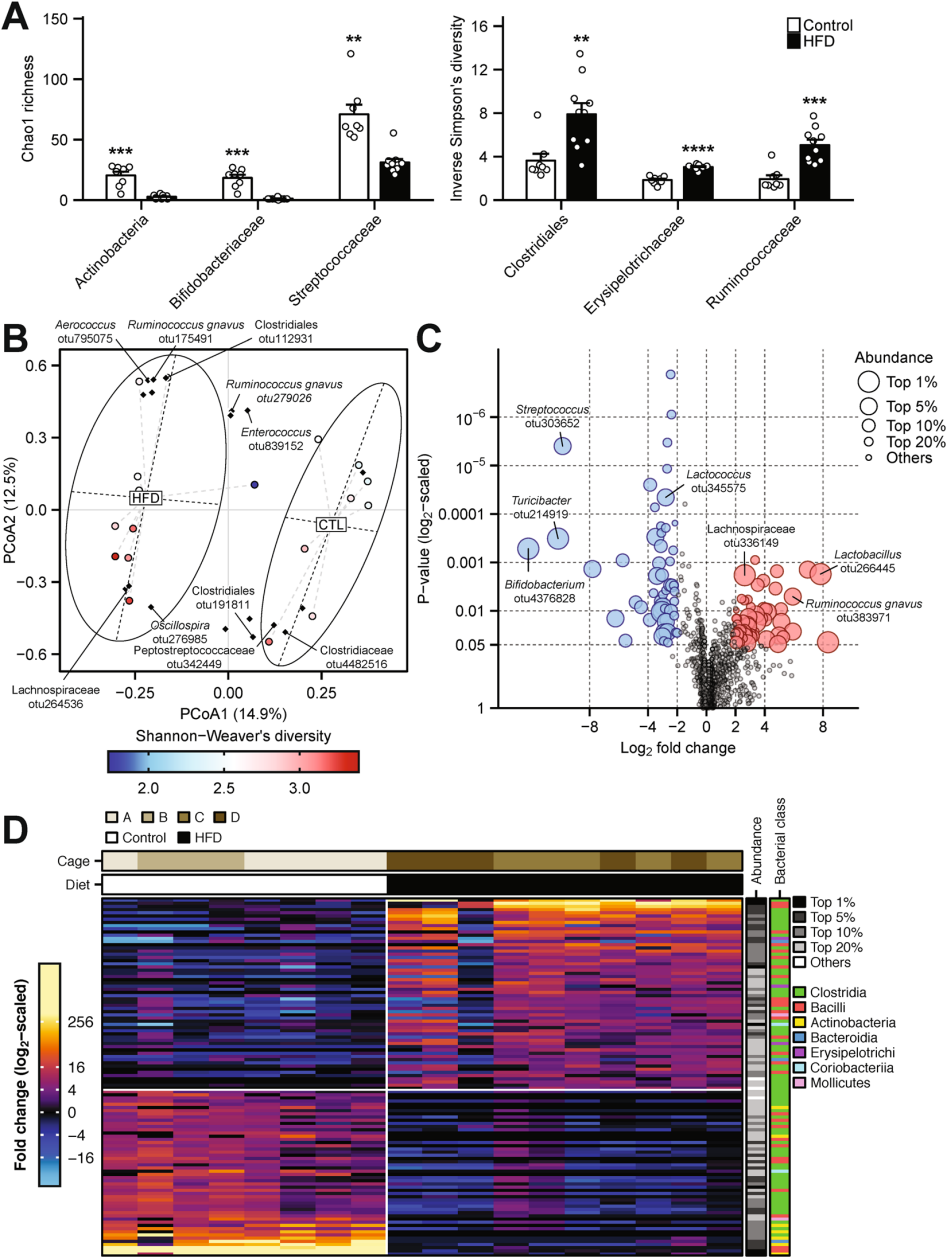
16S rRNA gene analysis of small intestinal microbiome. (**A**) α-diversity analysis of luminal microbiota operational taxonomic unit (OTU) at various taxonomic ranks. *p*-values are from two-tailed Student’s t-tests. (**B**) Principal coordinate analysis (PCoA) of β-diversity based on Bray-Curtis dissimilarity matrix of OTU-level compositional profiles. Ellipses represent 95% confidence intervals. Solid diamond-shaped points in black denote species scores, which were calculated using the *vegan* R-CRAN package. (**C**) Volcano plot showing relative trimmed-mean robust fold change in OTU abundance compared with the control group. *P*-values are computed by fitting multiple linear models with generalized least squares and derived by empirical Bayesian moderation of t-statistics on the fitted models as implemented in *limma* R/Bioconductor package. (**D**) Heatmap analysis of significantly enriched and depleted bacterial OTUs. **p < 0.01, ***p < 0.001, ****p < 0.0001 vs. control mice. Control, control group fed AIN-93M; HFD, HFD group fed HFD-60.

We performed subsequent experiments using HFD-treated small intestine prior to indomethacin administration. To investigate HFD-associated dysbiosis in the small intestine, the V3-V4 components of luminal microbiome samples were amplified and subjected to 16S rRNA gene sequence analysis. Comparative analysis of bacterial α-diversity metrics showed a reduced richness of operational taxonomic units (OTUs) in the class Actinobacteria (*p* = 6.3 × 10^−4^, false discovery rate [FDR] = 1.8 × 10^−2^), the families Bifidobacteriaceae (*p* = 3.1 × 10^−4^, FDR = 1.3 × 10^−2^), and Streptococcaceae (*p* = 1.2 × 10^−3^, FDR = 2.3 × 10^−2^), and increased OTU diversity in the order Clostridiales (*p* = 4.2 × 10^−3^, FDR = 5.9 × 10^−2^), and the families Erysipelotrichaceae (*p* = 1.1 × 10^−7^, FDR = 2.9 × 10^−6^), and Ruminococcaceae (*p* = 1.3 × 10^−4^, FDR = 2.7 × 10^−3^) in the HFD group (Fig. 2A). The overall richness and diversity of microbiota OTUs were not significantly different (Supplementary Fig. S2). Principal coordinate analysis (PCoA) of Bray-Curtis distances among the OTU profiles showed a clear separation between the HFD group and the control group (PERMANOVA, *p* = 1.0 × 10^−3^; Fig. 2B). Fold change analysis of individual OTUs suggested that the population of *Bifidobacterium* spp. decreased significantly in the HFD group (*p* = 1.0 × 10^−6^, FDR = 8.1 × 10^−5^; Fig. 2C). Heatmap visualization further demonstrated little sample variances in the depletion and enrichment of the bacterial OTU components across the dietary groups, which were independent of cage effects (Fig. 2D).

### Increase in intestinal permeability in HFD-fed mice

**Figure 3:**
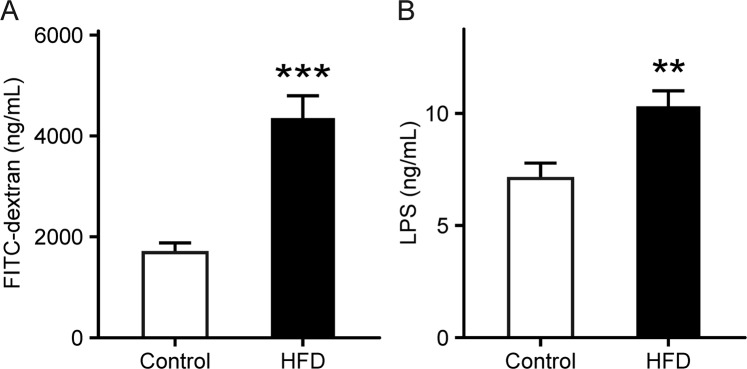
Gut leakiness in HFD-fed mice. (**A**) Intestinal permeability was examined by measuring serum FITC-dextran levels. n = 10. (**B**) Serum LPS levels were analyzed using the murine LPS ELISA kit. n = 8. Each column represents mean ± standard error of the mean. **p < 0.01, ***p < 0.001 vs. control mice. Control, control group fed AIN-93M; HFD, HFD group fed HFD-60.

Small intestinal permeability was examined by measuring fluorescein isothiocyanate (FITC)-dextran and serum lipopolysaccharide (LPS) that is a membrane component found in gram-negative bacteria. Serum FITC-dextran in the HFD group was significantly elevated by 2.0-fold prior to indomethacin administration compared with that in the control group (*p* = 3.3 × 10^−4^; Fig. 3A). This result was consistent with changes in serum LPS levels (*p* = 5.2 × 10^−3^; Fig. 3B).

### Effects of HFD treatment on the expressions of inflammatory cytokines and tight junction components

**Figure 4:**
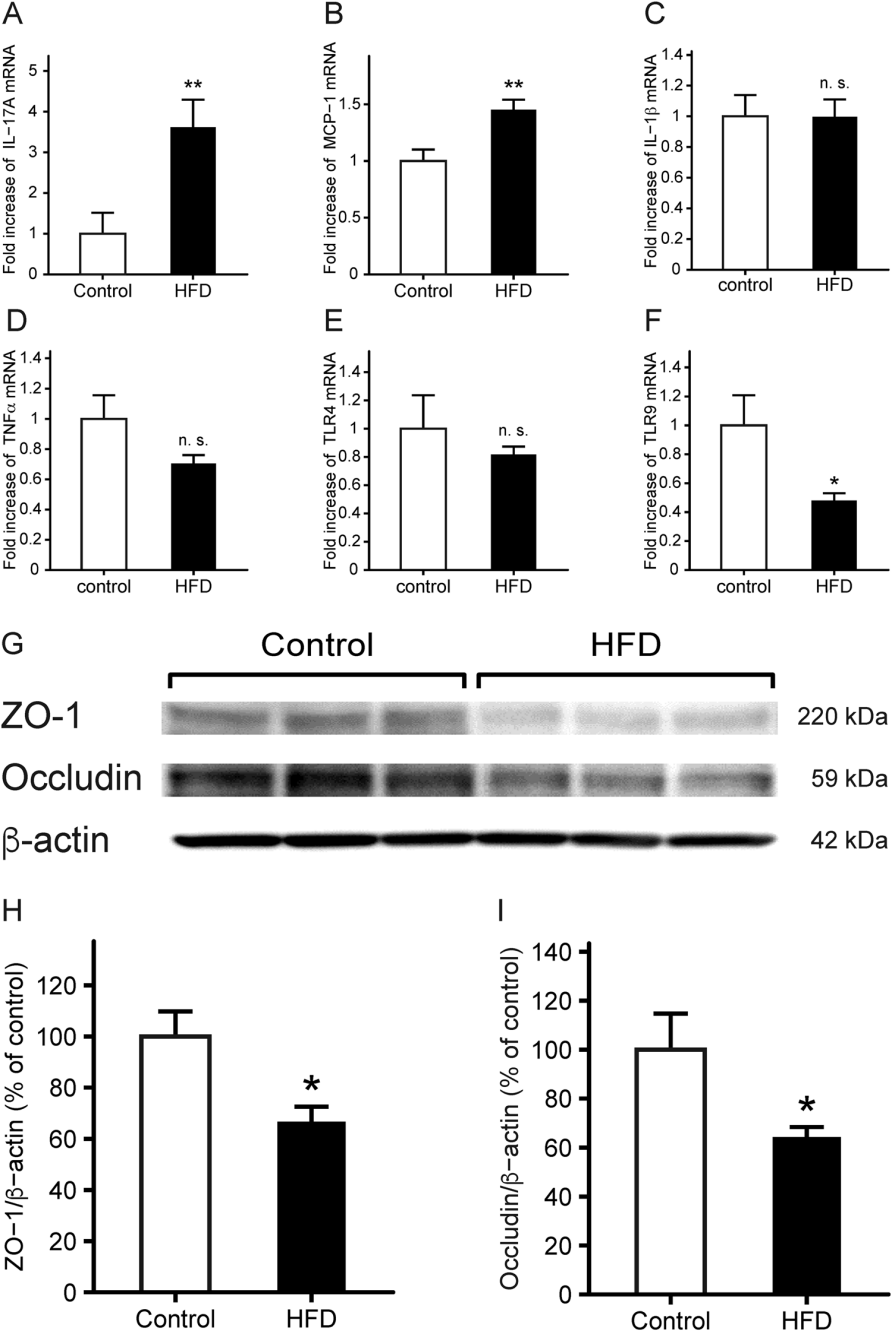
Small intestinal expressions of cytokines and tight junction components in HFD-fed mice. (**A**–**F**) Expressions of IL-17A (**A**), MCP-1 (**B**), IL-1β (**C**), TNF-α (**D**), TLR4 (**E**), and TLR9 (**F**) were evaluated by real-time quantitative RT-PCR. The mRNA levels are expressed as ratios of the mean value for control mice. n = 9–10. (**G**) Representative images of western blots for ZO-1 and occludin. Quantitative analyses of protein expressions for ZO-1 (**H**) and occludin (**I**) in the small intestine of HFD-fed mice. Protein levels are normalized to that of β-actin. n = 6–7. Each column represents mean ± standard error of the mean. *p < 0.05, **p < 0.01 vs. control mice. n. s., not significant; Control, control group fed AIN-93M; HFD, HFD group fed HFD-60.

We measured the expressions of inflammatory cytokines, innate immune receptors, and tight junction components in the small intestine of HFD-fed mice prior to indomethacin administration. The mRNA levels of interleukin (IL)-17A (Fig. 4A) and MCP-1 (Fig. 4B) in the HFD group were significantly increased by 3.5-fold (*p* = 7.8 × 10^−3^) and 1.5-fold (*p* = 3.3 × 10^−3^), respectively, although there were no differences in the expressions of other inflammatory cytokines, such as IL-1β, TNF-α, and toll-like receptor (TLR) 4 (Fig. 4C–E). The mRNA levels of TLR 9 decreased significantly in the HFD group (*p* = 3.8 × 10^−2^; Fig. 4F), and the expressions of tight junction proteins, such as ZO-1 (Fig. 4H) and occludin (Fig. 4I), were reduced in the small intestinal mucosa in the HFD group.

### Effects of neutralizing IL-17A on indomethacin-induced small intestinal damage in HFD-fed mice

**Figure 5:**
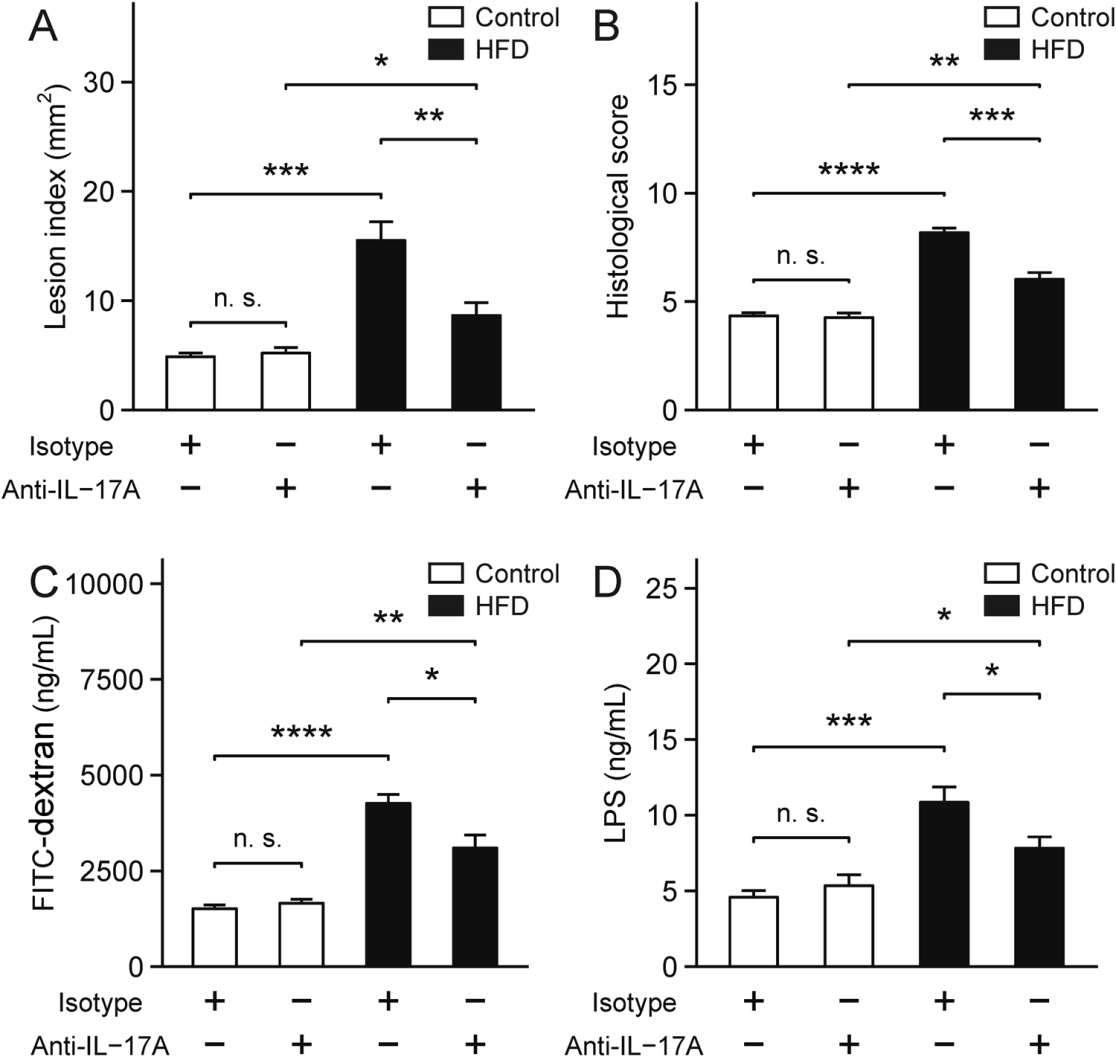
Effects of neutralization of IL-17A. (**A**) Lesion index of indomethacin-induced small intestinal damage. n = 7–10. (**B**) Histological evaluation of indomethacin-induced small intestinal damage. n = 5. (**C**) Intestinal permeability was examined by measuring serum FITC-dextran levels. n = 8–10. (**D**) Serum LPS levels were analyzed using the murine LPS ELISA kit. n = 9–10. Each column represents mean ± standard error of the mean. *p < 0.05, **p < 0.01, ***p < 0.001, ****p < 0.0001; n. s., not significant; Isotype, mice treated with isotype control; Anti-IL-17A, mice treated with anti-IL-17A antibody; Control, control group fed AIN-93M; HFD, HFD group fed HFD-60.

To determine the role of IL-17A, we administered anti-IL-17A antibody or isotype control to HFD-fed mice prior to indomethacin treatment once daily for 7 days. Two days after the final dose, we administered indomethacin (12 mg/kg BW) by gavage, and the mice were sacrificed 24 h later. Lesion index was significantly reduced in the HFD group treated with anti-IL-17A antibodies (*p* = 9.3 × 10^−3^; Fig. 5A), and this result was consistent with the histological observations (*p* = 8.0 × 10^−4^; Fig. 5B). Furthermore, IL-17A neutralization significantly reduced intestinal permeability and serum LPS in the HFD group (*p* = 1.4 × 10^−2^; Fig. 5C; *p* = 3.5 × 10^−2^; Fig. 5D).

### Transplantation of altered small intestinal microbiota from HFD-fed mice

**Figure 6:**
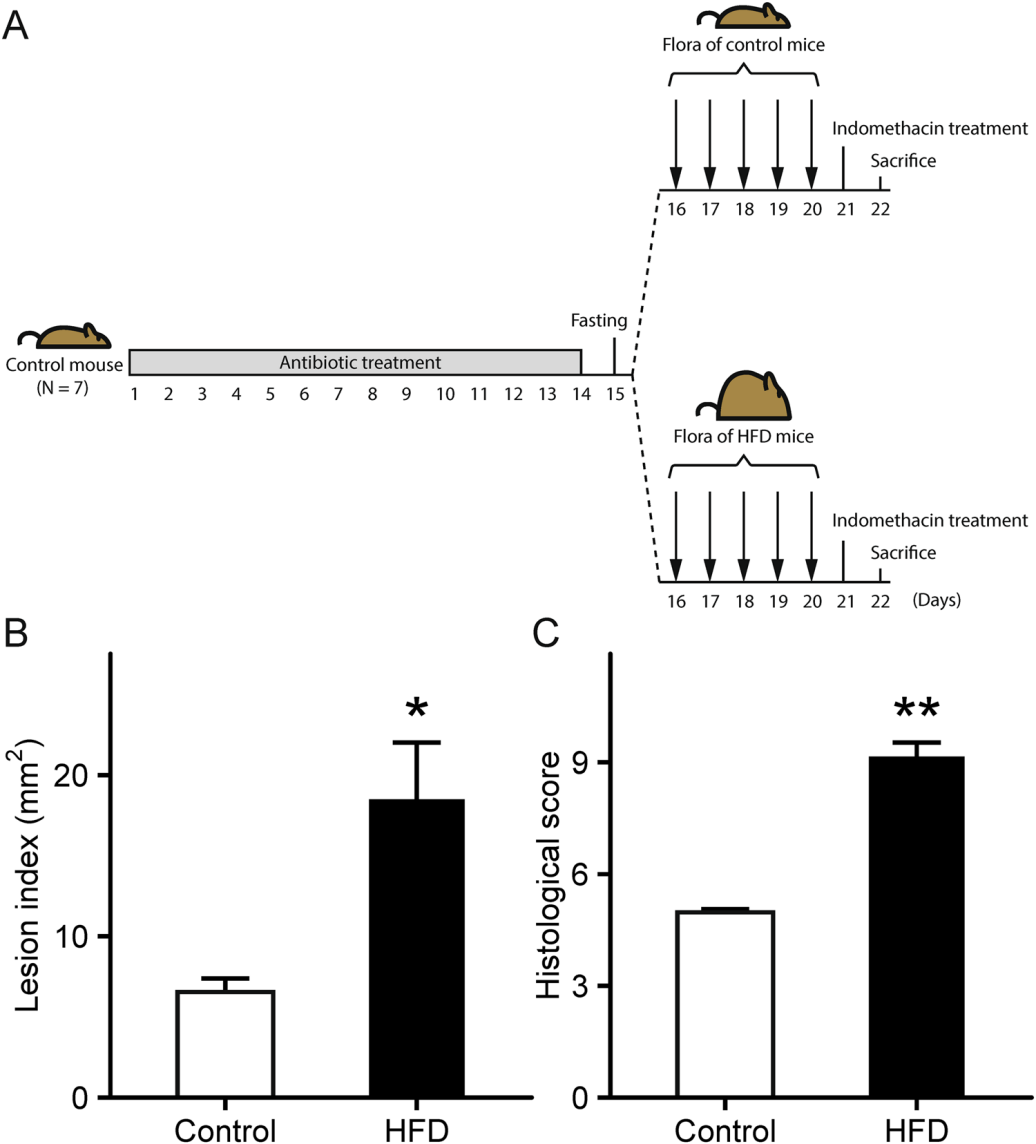
Effects of small intestinal microbiota transplantation. (**A**) Schematic timeline of small intestinal microbiota transplantation. (**B**) Lesion index of indomethacin-induced small intestinal damage after microbial transplantation from donor mice fed HFD-60 or AIN-93M. n = 7–8. (**C**) Histological evaluation of indomethacin-induced small intestinal damage after microbial transplantation from donor mice fed HFD-60 or AIN-93M. n = 4. Each column represents mean ± standard error of the mean. *p < 0.05, **p < 0.01 vs. control mice. Control, mice that received microbial transplantation from donor mice fed AIN-93M; HFD, mice that received microbial transplantation from donor mice fed HFD-60.

To investigate whether microbiota modulates indomethacin-induced small intestinal damage, small intestinal luminal contents collected from donor mice fed HFD-60 or AIN-93M were transplanted into microbiota-depleted recipient mice, treated with antibiotic cocktail, fed AIN-93M once daily for 5 days. One day after the final transplantation, mice were administered indomethacin (12 mg/kg BW) by gavage and sacrificed 24 h later (Fig. 6A). As a result, intestinal microbiota transplantation from HFD-60-fed mice exacerbated small intestinal damage, and significantly increased lesion index (*p* = 1.1 × 10^−2^; Fig. 6B) and histological score (*p* = 1.1 × 10^−3^; Fig. 6C) were observed.

## Discussion

In this study, we demonstrated that HFD treatment changed the gut microbiota in the small intestine and exacerbated NSAID-induced small intestinal damage. As the small intestinal microbiota plays an underlying role in the regulation of endotoxemia and inflammatory response, it is essential to identify major bacteria present in the small intestine of HFD-treated mice. Microbiota analysis showed that the proportion of gram-positive anaerobic bacilli, *Bifidobacterium* spp., was significantly reduced in HFD-fed mice. It has been reported that PPI-treatment exacerbated NSAID-induced small intestinal injury by inducing the reduction of *Bifidobacteria* spp., and recolonization with *Bifidobacteria*-enriched commensal bacteria prevented the damage^4^. *Bifidobacterium* spp. do not degrade intestinal mucus glycoproteins, but, instead, promote a stable environment and improve gut barrier function by inhibiting the translocation of pathogenic enterobacteria and endotoxin^11,12^. By contrast, increasing the proportion of gram-negative bacteria can decrease the integrity of intestinal mucosa and lead to metabolic endotoxemia^13^. In this study, it is assumed that a decrease in *Bifidobacterium* spp. was involved in intestinal hyperpermeability and metabolic endotoxemia observed in HFD-fed mice.

Intestinal permeability is regulated by the components of the epithelial cellular tight junction, such as ZO-1 and occludin. In this study, the protein expressions of ZO-1 and occludin decreased in the small intestinal mucosa of HFD-fed mice. The dysfunction of intestinal tight junction barrier is associated with intestinal microbiota dysbiosis^14^. Hence, we proposed that the disruption of intestinal tight junction components in this study was accelerated by HFD-induced dysbiosis, which exacerbated intestinal permeability. Consequently, endotoxin was increased in the blood, triggering a cascade of inflammatory responses. In a previous study, the administration of NSAID accelerated intestinal permeability during absorption^15^ and resulted in the induction of mucosal damage. In the present study, we assume that HFD-induced intestinal barrier dysfunctions facilitated enterobacterial invasion of mucosae and metabolic endotoxemia, which may have promoted the development of indomethacin-induced small intestinal damage.

We previously reported that when mucosal barrier function is disrupted, LPS and high-mobility group box 1 from the injured epithelial cells bind to TLR 4 and activate NF-κB, through the MyD88-dependent pathway and the NLR family pyrin domain-containing 3 inflammasome. This leads to the release of proinflammatory cytokines, such as TNF-α and IL-1β, in NSAID-induced small intestinal damage^16–18^. Although the mRNA levels of TLR4, TNF-α, and IL-1β did not change, the mRNA level of TLR9 decreased in small intestine treated with HFD before NSAID administration. TLR9 is involved in the activities of macrophages against bacterial infection^19^ by identifying unmethylated CpG-DNA sites in bacterial DNA^20^. It is considered a protective factor in intestinal homeostasis, and Western diets are associated with its dysfunction^21^. Long-term administration of HFD in this study may have reduced the expression of TLR9 resulting in the deactivation of its protective function in this study.

We demonstrated that IL-17A was elevated in HFD-treated small intestine, and the neutralization of IL-17A improved the indomethacin-induced damage, intestinal permeability, and characteristics of endotoxemia. IL-17A is a pro-inflammatory cytokine associated with a variety of inflammatory diseases^22^, and there are several reports indicating that HFD intake induces the expression of IL-17 and exacerbates inflammatory diseases, e.g., psoriasis, colitis, and steatohepatitis^23–25^. Recent studies suggested that HFD-induced microbial alterations, especially the expansion of Firmicutes^26,27^, might be responsible for IL-17 induction. It is possible that the increased populations of Clostridiales and Erysipelotrichaceae belonging to Firmicutes in the HFD group might have enhanced the expression of IL-17A, thereby promoting small intestinal microinflammation and intestinal permeability in this study. Our result aligns with that of a previous study, which demonstrated that gut epithelial barrier dysfunctions can be reversed by IL-17A neutralization in a neuro-psychoactive drug-induced bacterial infection model^28^. Therefore, a therapeutic approach targeting IL-17A may help to diminish the metabolic burden of endotoxemia caused by defective intestinal barrier functions. A deeper mechanistic understanding of how IL-17A may interact with microbiota for homeostatic control of intestinal epithelia is warranted for the development of a novel therapeutic strategy against NSAID-induced small intestinal damage.

In previous studies, it has been demonstrated that intestinal microbiota is involved in the pathogenesis of NSAID-induced enteropathy^29–31^. Therefore, drugs regulating intestinal flora, such as probiotics, can be utilized as therapeutic agents^32^. It is known that *Clostridium difficile*-induced colitis can be remarkably restored by changing intestinal flora through feces transplantation from healthy subjects into the large intestine of patients; hence, fecal microbiota transplantation (FMT) can be applied to various other intestinal diseases^33^. There is a scarcity of FMT studies on the flora of small intestine as most FMT studies have examined the bacterial flora of large intestine. In this study, small intestinal microbial transplantation from HFD-fed mice exacerbated indomethacin-induced small intestinal damage. The alterations of small intestinal microbiota with HFD treatment may have increased the susceptibility of mucosae and they were sufficient to influence on indomethacin-induced enteropathy. To the best of our knowledge, this is the first report demonstrating that HFD-induced dysbiosis forms the substrate to aggravate indomethacin-induced enteropathy through IL-17A.

In conclusion, HFD-induced alterations of small intestinal microbiota cause microinflammation through the induction of IL-17A, and an increase in intestinal permeability, which aggravate NSAID-induced small intestinal damage. Low-fat dietary therapy and strategies to enhance individual-specific innate functions of *Bifidobacterium* may be useful for the prevention and mitigation of NSAID-induced small intestinal damage. A therapeutic strategy targeting IL-17A could be a treatment option for such enteropathy.

## Methods

### Animals and diets

Specific pathogen-free male C57BL/6 J mice (4 weeks old, 10–15 g) were purchased from Charles River Japan Inc. (Atsugi, Japan). Animals were housed in polycarbonate cages with paper-chip bedding in an air-conditioned biohazard room with a 12-h light/dark cycle. All animals had free access to food and water. Mice were acclimated for 1 week prior to the experiments and received regular chow (CE-2, CLEA Japan Inc., Tokyo, Japan). At 6 weeks of age, mice were randomly separated into a control group fed AIN-93M (Oriental Yeast Co., Tokyo, Japan) or an HFD group fed HFD-60 (Oriental Yeast Co.) for 8 weeks. The composition of AIN-93M and HFD-60 is shown in Supplementary Table S1.

All experiments were carried out under the control of animal research committee in accordance with the Guidelines on Animal Experiments in Osaka City University Graduate School of Medicine, the Japanese Government Animal Protection and Management Law (No. 105), and the Japanese Government Notification on Feeding and Safekeeping of Animals (No. 6). All experimental procedures were approved by the Animal Care Committee of Osaka City University Graduate School of Medicine (Approval number 16014). All surgeries were performed under isoflurane, with maximal efforts taken to minimize suffering.

### Induction of experimental small intestinal damage

Non-fasted mice were administered indomethacin (12 mg/kg BW; Sigma-Aldrich, St Louis, MO, USA) in 0.5% carboxymethylcellulose solution by gavage and were sacrificed 24 h later. Small intestinal damage was assessed using 1% Evans blue by intravenous injection 30 min before sacrifice. The small intestine was opened along the antimesenteric attachment and examined for damage under a dissecting microscope with square grids (×10). The area (mm^2^) of macroscopically visible lesion was measured, summed per small intestine, and used as the lesion index. Prior to indomethacin treatment, tissue samples with luminal contents were collected (4.5 cm in length each) from lower ileum section at a position approximately 2 cm proximal to the terminal ileum. Blood samples were obtained by cardiac puncture.

### Histological examination of small intestinal damage

Tissue samples were fixed in 0.1 M phosphate buffer (pH 7.4) containing 4% paraformaldehyde and embedded in OCT compound (Miles, Elkhart, IN, USA). Serial 5-μm-thick cryostat sections were mounted on silanized slides (Dako, Tokyo, Japan) and stained with hematoxylin and eosin. For each mouse, at least ten random villi at the damaged areas were scored in a masked fashion by 2 investigators independently. For quantitative evaluation, a modified histological scoring system was used^18^. Total histology scores ranged from 0 to 13 and were evaluated for 6 categories with sub-scores as follows: epithelium (0, normal; 1, flattened; 2, loss of epithelial continuity; 3, severe denudation), villus shape (0, normal; 1, short and rounded; 2, extremely short and thick), villus tip (0, normal; 1, damaged; 2, severely damaged), stroma (0, normal; 1, slightly retracted; 2, severely retracted), inflammation (0, no infiltration; 1, mild infiltration; 2, severe infiltration), and crypt status (0, normal; 1, mild crypt loss; 2, severe crypt loss).

### DNA extraction and 16S rRNA gene analysis

Small intestinal luminal contents were collected immediately after sacrifice, using a feces collection kit (Takara Bio, Shiga, Japan). Genomic DNA was then isolated using the NucleoSpin Microbial DNA Kit (MACHEREY-NAGEL, Düren, Germany). Approximately 500 µl of stored small intestinal samples were placed in a microcentrifuge tube containing 100 µl elution buffer. The mixture was placed into the NucleoSpin Beads Tube with proteinase K, which was subjected to mechanical beads-beating for 12 min at 30 Hz in the TissueLyzer LT. DNA extraction was subsequently performed as per manufacturer’s instructions. DNA samples were purified using the Agencourt AMPure XP (Beckman Coulter, Brea, CA).

V3–4 amplicon sequences of 16S rRNA genes were prepared as described previously^34^. Briefly, 16S rRNA gene fragments including V3 and V4 regions were amplified by PCR (forward primer: ACACGACGCTCTTCCGATCTCCTACGGGNGGCWGCAG, reverse primer: GACGTGTGCTCTTCCGATCTGACTACHVGGGTATCTAATCC) for 20 cycles, and PCR products were purified with Agencourt AMpure beads (Beckman Coulter). Next, index sequences for sequencing were added by running a second PCR with NEBNext Multiplex Oligos for Illumina (Dual Index Primers Set1, New England Biolabs) for 8 cycles, and the products were purified with Agencourt AMpure beads. For each sample, an equal amount of each DNA amplicon library was mixed and sequenced on the MiSeq instrument (Illumina) using a MiSeq v3 Reagent kit with 20% PhiX (Illumina).

### FITC-Dextran permeability assay

Intestinal permeability was measured as previously described with some modifications^10^. Mice were fasted for 16 h and FITC-dextran was administered by gavage (500 mg/kg BW, 125 mg/ml; Sigma-Aldrich). After 4 h of administration, 120 μl of blood was collected from the heart, stored on ice in the dark, and centrifuged (15,000 rpm) at 4 °C for 10 min. Serum was diluted in an equal volume of PBS (pH 7.4) and analyzed for FITC-dextran concentration with a fluorescence spectrophotometer (Varioskan LUX; Thermo Scientific, Waltham, MA, USA) at an excitation wavelength of 485 nm and an emission wavelength of 535 nm.

### Blood sampling and LPS assay

Blood samples were obtained by cardiac puncture and stored on ice for 30 min before centrifugation (15,000 rpm) at 4 °C for 10 min. Serum portions were isolated, and LPS levels were measured with the murine LPS ELISA kit (Cusabio, Wuhan, China).

### Real-time quantitative RT-PCR

Total RNA was extracted from small intestinal tissues with the ISOGEN kit (Nippon Gene Co., Ltd., Tokyo, Japan). Real-time quantitative RT-PCR analyses were performed as previously described^35^. The sequences of PCR primers and TaqMan probes of ZO-1, MCP-1, IL-1β, TNF-α, and TLR 4 are shown in Supplementary Table S2. TaqMan Gene Expression Assays (Life Technologies Corp., Rockville, MD, USA) were used for measuring occludin, IL-17A, and TLR9. Glyceraldehyde-3-phosphate dehydrogenase (GAPDH) was used as an internal control, and mRNA levels were expressed as ratios relative to the mean of control mouse data.

### Western blotting

Small intestinal tissues were homogenized and lysed on ice in buffer containing 0.5% NP-40, 40 mM Tris-HCl (pH 8.0), 120 mM NaCl, 1 mM PMSF, and 10 μg/ml leupeptin. Proteins in lysates were measured with a modified bicinchoninic acid method. Proteins were denatured with SDS sample buffer, subjected to 10% SDS-polyacrylamide gel electrophoresis, and transferred to PVDF membrane. Membranes were blocked with TBS buffer (10 mM Tris-HCl, pH 7.5, 100 mM NaCl, and 0.1% Tween-20) containing 5% BSA and were incubated overnight with rabbit polyclonal antibodies against ZO-1 (catalog no. 61–7300, Invitrogen, Carlsbad, CA, USA; diluted 1:1000), or rabbit monoclonal antibodies against occludin (catalog no. ab168986, Abcam, Cambridge, UK; diluted 1:1000). Antigen-antibody complexes were detected with anti-rabbit IgG-HRP (diluted 1:1000) using enhanced chemiluminescence in accordance with the manufacturer’s instructions (Amersham, Arlington Heights, IL, USA). Bands were quantified using laser-scanning densitometry and the expression level of each protein was normalized against that of β-actin. Full-length Western blots of ZO-1 and occludin are shown in Supplementary Fig. S3.

### Neutralization of IL-17A

After 8 weeks on control diet or HFD, mice were subjected to intraperitoneal injection of anti-IL-17A antibody (4 mg/kg; clone 17F3, mouse IgG1, BioXCell, West Lebanon, NH, USA) or mouse IgG1 isotype control (4 mg/kg; clone MOPC-21, mouse IgG1, BioXCell) once daily for 7 days. Two days after the final administration of antibodies, mice were administered indomethacin (12 mg/kg; Sigma-Aldrich) in a 0.5% carboxymethylcellulose solution by gavage and sacrificed 24 h later.

### Small intestinal microbiota transplantation

After 8 weeks on control diet, recipient mice were given drinking water containing ampicillin (1 g/l), metronidazole (1 g/l), neomycin (1 g/l), and vancomycin (0.5 g/l) for 2 weeks. Small intestinal luminal contents from donor mice were collected, dissolved in 10 g/L sterile PBS, centrifuged at 1,500 rpm for 5 min. Supernatants were administered to the recipient mice by gavage (0.5 ml/mouse) for 5 days. After the final day of transplantation, mice were administered indomethacin (12 mg/kg BW; Sigma-Aldrich) in a 0.5% carboxymethylcellulose solution by gavage and sacrificed 24 h later.

### Statistical analysis

Values are expressed as mean ± standard error of the mean (SEM) in animal experiments. Comparisons between 2 groups were performed using a two-sided Student’s *t*-test. ANOVA was used to compare differences among multiple groups, and post-hoc analysis was performed by Tukey’s multiple comparisons test. *P*-values < 0.05 indicate a statistical significance.

## Supplementary information

Supplementary file
