# Supplementary material for: High-fat diet-mediated dysbiosis exacerbates NSAID-induced small intestinal damage through the induction of interleukin-17A
Source: Sci Rep. 2019 Nov 14;9:16796. doi: 10.1038/s41598-019-52980-2 (PMC6856170; doi:10.1038/s41598-019-52980-2)
Supplement: Supplementary file 1 — Supplementary file [file 41598_2019_52980_MOESM1_ESM.pdf]

High-fat diet-mediated dysbiosis exacerbates NSAID-induced small intestinal damage through the induction of interleukin-17A

Naoki Sugimura[1], Koji Otani[1], Toshio Watanabe[1*], Geicho Nakatsu[3], Sunao Shimada[1], Kosuke Fujimoto[2], Yuji Nadatani[1], Shuhei Hosomi[1], Fumio Tanaka[1], Noriko Kamata[1], Koichi Taira[1], Yasuaki Nagami[1], Tetsuya Tanigawa[1], Satoshi Uematsu[2], Yasuhiro Fujiwara[1]

[1]Department of Gastroenterology, Osaka City University Graduate School of Medicine, 1-4-3 Asahimachi, Abeno-ku, Osaka 545-8585, Japan
[2]Department of Immunology and Genomics, Osaka City University Graduate School of Medicine, 1-4-3 Asahimachi, Abeno-ku, Osaka 545-8585, Japan; Division of Innate Immune Regulation, International Research and Development Center for Mucosal Vaccines, The Institute of Medical Science, The University of Tokyo, 4-6-1 Shirokanedai, Minato-ku, Tokyo 108-8639, Japan
[3]Department of Immunology and Infectious Diseases/Genetics and Complex Diseases, Harvard T. H. Chan School of Public Health, Room 904, Building 1, 665 Huntington Avenue, Boston, Massachusetts 02115, United States

*Corresponding Author: Toshio Watanabe
Department of Gastroenterology, Osaka City University Graduate School of Medicine, 1-4-3 Asahimachi, Abeno-ku, Osaka 545-8585, Japan
Tel: +81-6-6645-3811
Fax: +81-6-6645-3813
E-mail: watanabet@med.osaka-cu.ac.jp

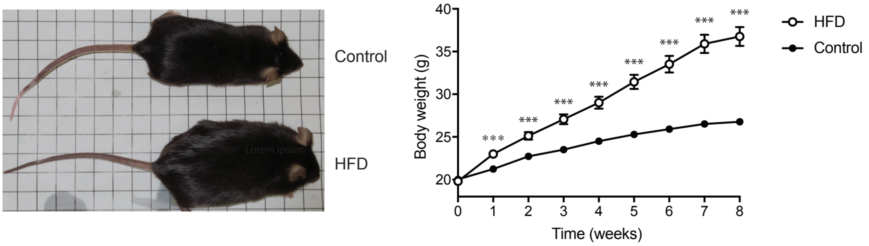

Supplementary Figure S1. Body weight changes. Body weight changes in male C57BL/6J mice fed high-fat diet or normal diet for 8 weeks. n = 15. ***p < 0.001 vs. control mice. Control, control group fed AIN-93M; HFD, HFD group fed HFD-60.

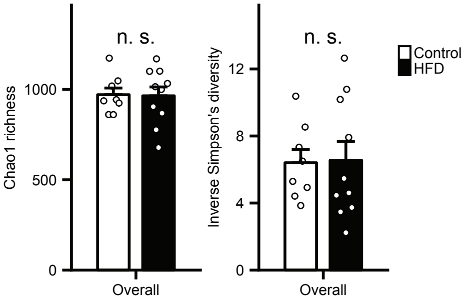

Supplementary Figure S2. α-diversity analysis. α-diversity analysis of overall small intestinal bacterial OTU compositions in control and HFD-fed mice. n. s., not significant. Control, control group fed AIN-93M; HFD, HFD group fed HFD-60.

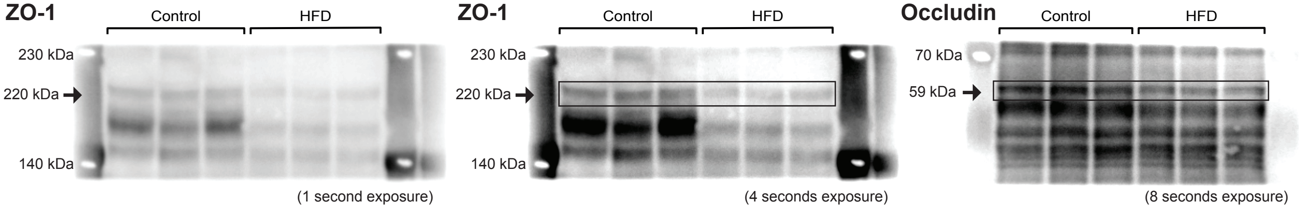

Supplementary Figure S3. Full-length Western blots of ZO-1 and Occludin. The bands for ZO-1 and Occludin marked by black boxes are shown in Figure 4.

	AIN-93M	HFD-60
Calorie (kcal/g)	3.8	5.1
Fat (%)	3.9	35.0
Protein (%)	12.5	23.0
Carbohydrate (%)	69.1	23.5
Others (%)	14.5	18.5

Supplementary Table S1. Diet composition.

Gene	Primers and probes	
ZO-1	Primer (forward)	5'-GAGGAAACAGCTATATGGGAACAG-3'
	Primer (reverse)	5'-CCCTCCTTTTAACACATCAGAAATCA-3'
	Probe	5'-FAM-CAGTGACGCTTCACAGGGCTCCTGG-TAMRA-3'
MCP-1	Primer (forward)	5'-GCTGCTACTCATTCACTGGCAA-3'
	Primer (reverse)	5'-TGCTGCTGGTGATTCTCTTGTA-3'
	Probe	5'-FAM-ATGATCCCAATGAGTCGGCTGGAGA-TAMRA-3'
IL-1β	Primer (forward)	5'-ACAGGCTCCGAGATGAACAAC-3'
	Primer (reverse)	5'-CCATTGAGGTGGAGAGCTTTC-3'
	Probe	5'-FAM-GAAAAAGCCTCGTGCTGTCGGACCCATAT-TAMRA-3'
TNF-α	Primer (forward)	5'-TCATGCACCACCATCAAGGA-3'
	Primer (reverse)	5'-GAGGCAACCTGACCACTCTCC-3'
	Probe	5'-FAM-AATGGGCTTTCCGAATTCACTGGAGC-TAMRA-3'
TLR4	Primer (forward)	5'-CATGGAACACATGGCTGCTAA-3'
	Primer (reverse)	5'-CCCCTGGAAAGGAAGGTGTC-3'
	Probe	5'-FAM-TATAGCATGGACCTTACCGGGCAGAAGG-TAMRA-3'

Supplementary Table S2. PCR primers and TaqMan probes.
